# The neglected avian hepatotropic virus induces acute and chronic hepatitis in ducks: an alternative model for hepatology

**DOI:** 10.18632/oncotarget.19003

**Published:** 2017-07-05

**Authors:** Xumin Ou, Sai Mao, Jingyu Cao, Yunchao Ma, Guangpeng Ma, Anchun Cheng, Mingshu Wang, Dekang Zhu, Shun Chen, Renyong Jia, Mafeng Liu, Kunfeng Sun, Qiao Yang, Ying Wu, Xiaoyue Chen

**Affiliations:** ^1^ Institute of Preventive Veterinary Medicine, Sichuan Agricultural University, Wenjiang, Chengdu, Sichuan, People′s Republic of China; ^2^ Key Laboratory of Animal Disease and Human Health of Sichuan Province, Sichuan Agricultural University, Wenjiang, Chengdu, Sichuan, People′s Republic of China; ^3^ Avian Disease Research Center, College of Veterinary Medicine, Sichuan Agricultural University, Wenjiang, Chengdu, Sichuan, People′s Republic of China; ^4^ China Rural Technology Development Center, Beijing, People′s Republic of China

**Keywords:** duck hepatitis A virus, viral hepatitis, liver injury, pathogenesis, viral-host interaction, Pathology Section

## Abstract

Duck Hepatitis A Virus (DHAV) belongs to the *Avihepatovirus*, which is also classified into *Picornaviridae* with *Hepatovirus*, Hepatitis A Virus (HAV). In humans, the pathogenesis of HAV is not well understood because of limited work with animal models. Here, we investigated the progress of duck viral hepatitis caused by DHAV and their potential for dissecting the pathogenesis of HAV. During the course of infection, the duck model had undergone hepatocellular lesions (vacuolation, acidophilic degeneration and steatosis), lymphocytes recruitment (neutrophil granulocytes, heterophilic granulocytes and T cells or plasm cells) and repair (activation of hepatic stellate cells, fibrosis and regeneration). Coincident with liver injury, the serum biomarkers, aspartate aminotransferase and alanine transaminase were significantly increased. Moreover, comparatively lower CD4+ and CD8+ T-cells were recruited to the liver, which might lead to a persistent infection (40 wk). Because DHAV and HAV have similar genomic structure, biological phenotypes and can easily replicate in liver. And half of fibrosis-related genes had high homology between humans and ducks. Considering these similarity in pathological and virological phenotypes, we proposed that the ducks might be an alternatively small animal model that would provide insight into the pathogenesis of viral hepatitis, fibrosis and liver regeneration.

## INTRODUCTION

Hepatitis A is a worldwide human liver disease caused by Hepatitis A Virus (HAV), especially in areas of lower socio-economic status and reduced sanitary conditions. HAV, a member of the *Hepatovirus* genus in the family of *Picornaviridae*, has an estimated 1.4 million cases occur each year [1]. However, there is a similar liver disease in ducks caused by Duck Hepatitis A virus (DHAV) [2, 3]. DHAV, a member of the *Avihepatovirus* genus within the same family *Picornaviridae*, causes a highly fatal, rapidly infectious disease in ducklings, characterized by swelling livers mottled with haemorrhages [3-6]. This type of viral hepatitis in ducklings should be classified as acute viral hepatitis, but the progressive pathogenesis caused by DHAV in mature ducks has been largely unexplored.

Chronic Viral Hepatitis (CVH) is mainly caused by Hepatitis B Virus (HBV), Hepatitis C Virus (HCV), and Hepatitis D Virus (HDV), but humans infected with HAV do not progress to CVH [7-10]. Briefly, CVH is transformed from Acute Viral Hepatitis (AVH) and is related to inflammatory responses, parenchymal cell necrosis or apoptosis and regeneration. Many human and animal model studies have revealed a correlation between inflammatory responses and fibrosis, which is caused by proliferation of hepatic stellate cells (HSCs) [11, 12]. Additionally, approximately 5%-10% of adult patients with CVH may progress to liver cirrhosis [13]. Hepatocytes are the major sources of matrix metalloproteinases (MMP), and their inhibitors are involved in liver extracellular cell matrix (ECM) deposition, which is involved in the pathogenesis of liver cirrhosis in CCL_4_-induced liver cirrhosis in rats [14]. The endothelial fenestrae is 150-175 nm in diameter and acts as a dynamic filter facilitating the exchange of nutrition and particles between parenchymal cells and the hepatic sinusoid. Many hepatitis viruses could result in defenestration and a decrease in the number of fenestrae [15]. Apoptosis or necrosis is the most common consequence of hepatocellular injury, followed by KCs engulfing apoptotic bodies and death ligands, including the Fas ligand and TNF-α, thereby promoting inflammation and fibrogenesis [16, 17]. Damaged hepatocytes will then induce activation of HSCs to stimulate the fibrogenic actions through release of reactive oxygen species (ROS) and fibrogenic mediators [18]. Hepatocyte apoptosis was also associated with expression of death receptors and activation of NF-κB in patients with steatohepatitis [19]. In addition, plentiful cytokines were involved in regulation of liver fibrosis, and most of them were widely used to attenuate the apoptotic and fibrotic effects [9, 15, 18, 20]. IFN-α was shown to reduce apoptotic effects on activated HSCs, and HSCs were also inactivated by IFN-β and then decreased the synthesis of α-smooth muscle actin (SMA) and collagen through inhibition of the TGF-β and PDGF pathways [21]. Moreover, the activation of HSCs is also inhibited by IFN-γ to decrease ECM deposition through inhibiting TGFβ1/Smad3 signalling pathways [22, 23]. On the contrary, interleukin 1 (IL-1), a proinflammatory cytokine, produced by KCs and sinusoidal endothelial cells (SECs) can activate HSCs to produce MMP-9, MMP-13 and TIMP-1, resulting in liver fibrogenesis [11]. IL-6 can attenuate hepatocyte apoptosis and promote their regeneration through NF-κB signalling and the Ras-MAPK pathway [24, 25].

Due to the crucial role of the animal model in understanding the pathogenesis and the development of therapeutic strategies for liver injury, fibrosis and cirrhosis, many types of mammal models have been developed in mice, rats, chimpanzees, rabbits, pigs and horses to mimic the process of liver injury [26-28]. However, owing to the proposed listing of the endangered primates and the inability to include them in research, optional duck models are essential, much more accessible, easier for laboratory management and also available for serial sampling of tissue in the volume required for detailed studies [29]. Previously, ducks have been widely used in Hepatitis B Virus preclinical trials and to elucidate their life cycle [30, 31]. Duck Hepatitis B Virus (DHBV ) infection cannot induce apparent liver injury and will process to hepatocellular carcinoma (HCC) [32]. However, avian hepatotropic virus (DHAV) can cause obvious liver disease characterized by swelling livers mottled with haemorrhages in ducklings. In this study, the course of hepatitis and host immune responses in mature ducks induced by DHAV were investigated to mimic the process of liver injury, fibrosis and regeneration.

## RESULTS

### Post-infection with DHAV in mature ducks was associated with acute and chronic hepatitis

**Figure 1:**
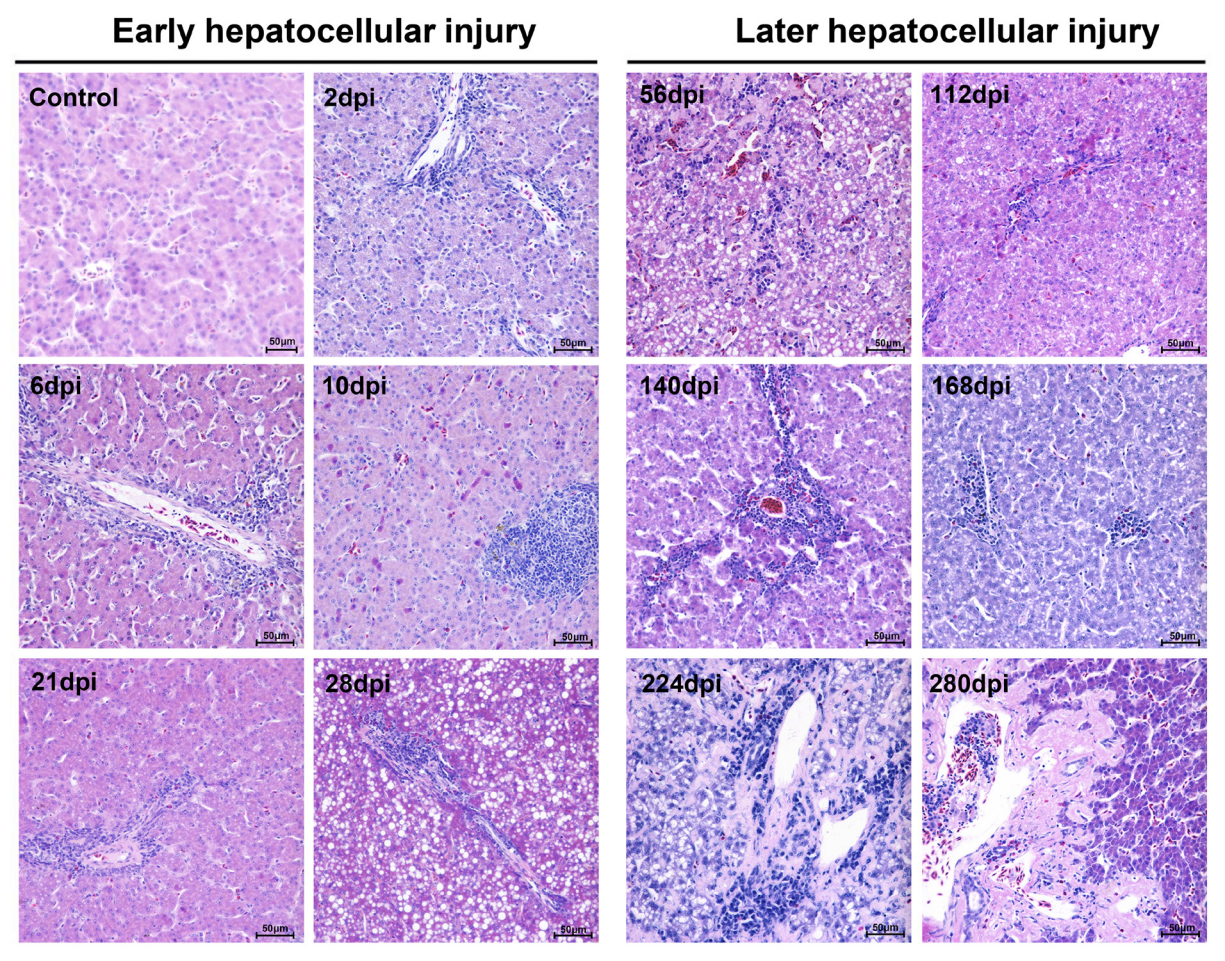
Microscopic lesions in mature ducks experimentally infected with DHAV-1 H strain Representative liver HE staining from 1d to 280d post-infection with DHAV are displayed from two stages, early infection and later infection (*n* = 5). (Con) represents pre-inoculation liver photomicrograph. The brightness and contrast are slightly modified to create a uniform background.

Liver tissues from infected breeding ducks had developed apparent liver injury with acute hepatitis at early infection and chronic hepatitis at later infection characterized by histopathological changes. At the early stage of infection, liver lesions were characterized by acidophilic degeneration, vacuolation and infiltration of neutrophil granulocytes (Figure 1). The liver had also developed apparent serious steatosis at 28-dpi (Figure 1). However, at the later stage of infection, acidophilic degeneration disappeared instead of serious vacuolation (ballooning degeneration). The intensity of steatosis, interface hepatitis and neutrophil granulocytes were temporarily elevated and gradually decreased in the last stage of infection. Of note, the changes of knodell Histological Activity Index (HAI) was highly coincident with the severity of liver injury (Table 1). Many more heterophilic granulocytes and plasma cells were strongly recruited to the liver until the occurrence of liver cell regeneration. Accompanying serious liver injury and inflammatory response, the fibrosis secreted by fibroblasts also took place from 10-dpi to the last stage of infection to make up the hepatocellular lesions (Supplementary Figure 1).

**Table 1:** The hepatitis activity during the course of infection.

Phases Factors	Early hepatocellular injury						Later hepatocellular injury					
	Con	2d	6d	10d	21d	28d	56d	112d	140d	168d	224d	280d
**I, Periportal +/-bridging necrosis**	0	1	1	1	1	1	3	4	5	4	5	1
**II, Intralobular degeneration and focal necrosis**	0	1	1	3	4	4	4	4	4	4	4	1
**III, Portal inflammation**	0	1	3	3	3	3	4	4	4	3	4	1
**IV, Fibrosis**	0	0	0	1	1	3	1	1	1	3	4	4
**Total scores**	0	3	5	7	9	11	12	13	14	14	17	7

The hepatitis activity scored by knodell Histological Activity Index (HAI) score system. The total score was calculated by four indexes, such as periportal +/- bridging necrosis, intralobular degeneration and focal necrosis, portal inflammation and fibrosis. The HAI scores were assessed by three diagnose pathologists.

**Figure 2:**
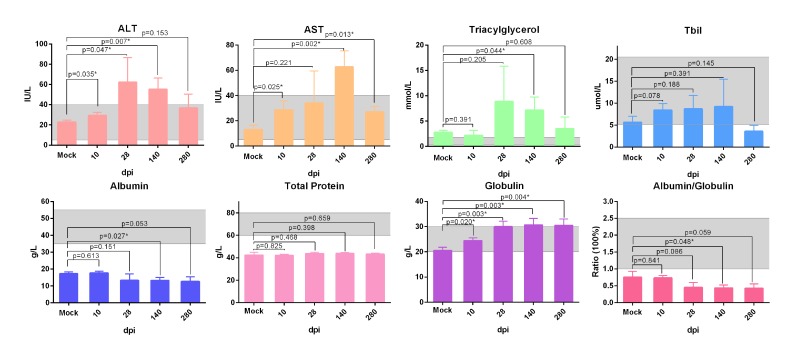
Biochemical detection of serum from ducks infected with DHVA-H Serum biochemical markers, ALT, AST, triglyceride, total Bilrubin, total protein, albumin, globulin and ratio of albumin and globulin, were analysed by auto biochemical detector. The scope between two dash lines was the normal biochemical reference range in human. Data are presented as means ± SD (*n* = 3). *P < 0.05 indicates significant difference using student’s T-test.

Additionally, the biochemical analysis of serum from infected ducks indicated that serum aspartate aminotransferase (AST), alanine transaminase (ALT) and total bilirubin (T-Bil) were increased and decreased successively in the course of infection (*P* < 0.05 ) (Figure 2). Moreover, the highly elevation of triacylglycerol levels at 28dpi was accord with serious steatosis identified by HE staining (Figure 1, 2). The significantly decreased albumin indicated the liver protein synthesis was seriously impaired during the infection. Simultaneously, the increased globulin indicated humoral immunity might not be impaired by DHAV (Figure 2).

### Comparatively lower T-cells in liver were associated with chronic infection

**Figure 3:**
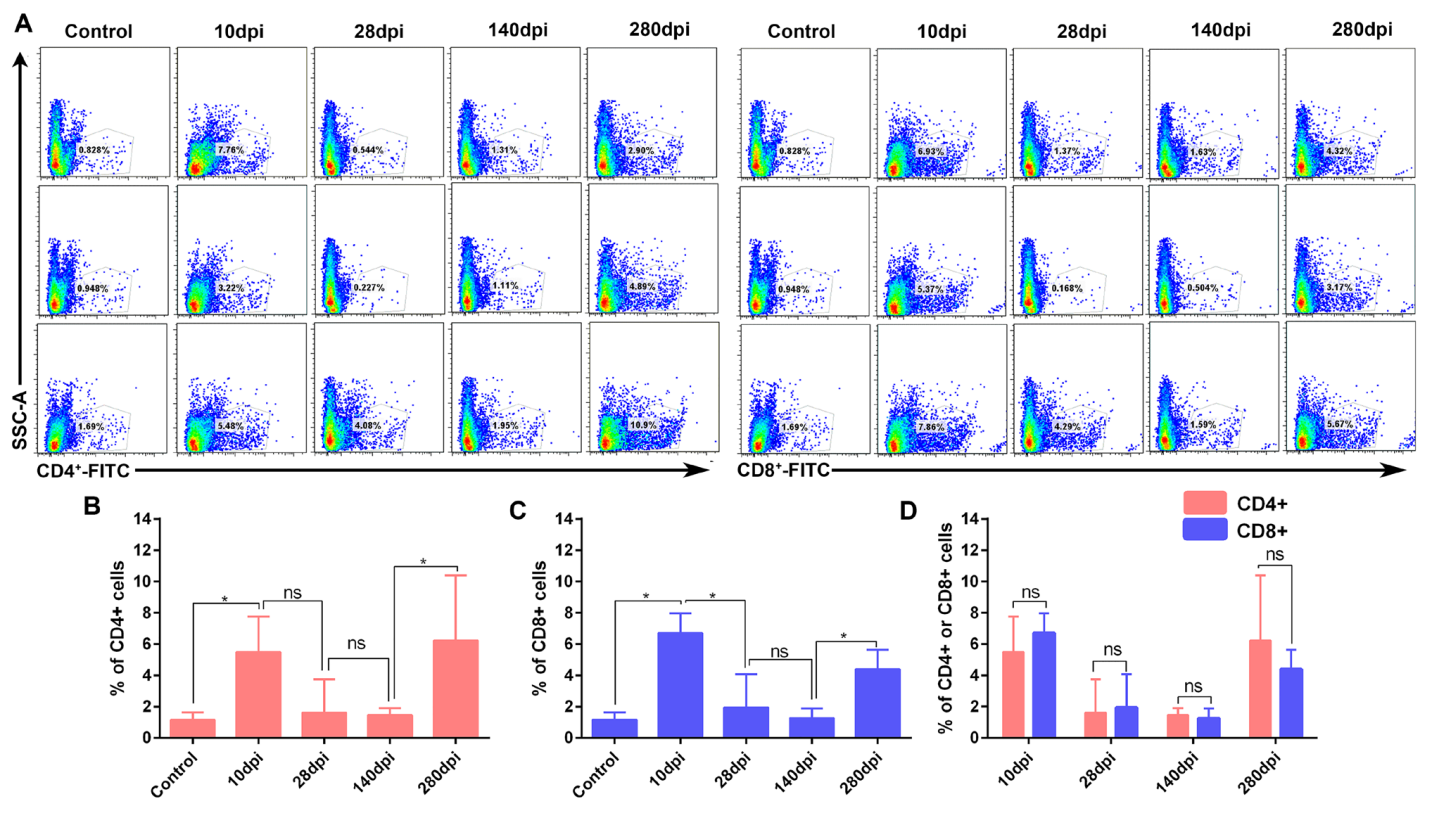
Flow cytometry analysis of CD4+ and CD8+ T-cells in liver (n = 3) The percentage of CD4+ and CD8+ T-cells were detected by FITC-conjugated goat anti mouse IgG. **A.** Flow cytometry analysis of CD4+ and CD8+ T-cells from 10 dpi to 280dpi. **B.** Significant difference analysis of positive CD4+ T cells in the course of infection. **C.** Significant difference analysis of positive CD8+ T cells in the course of infection. **D.** Significant difference analysis of between positive CD4+ and CD8+ T cells in the course of infection. Data are presented as means ± SD (n = 3). *P < 0.05 indicates significant difference using student’s T-test. ns, no significance.

**Figure 4:**
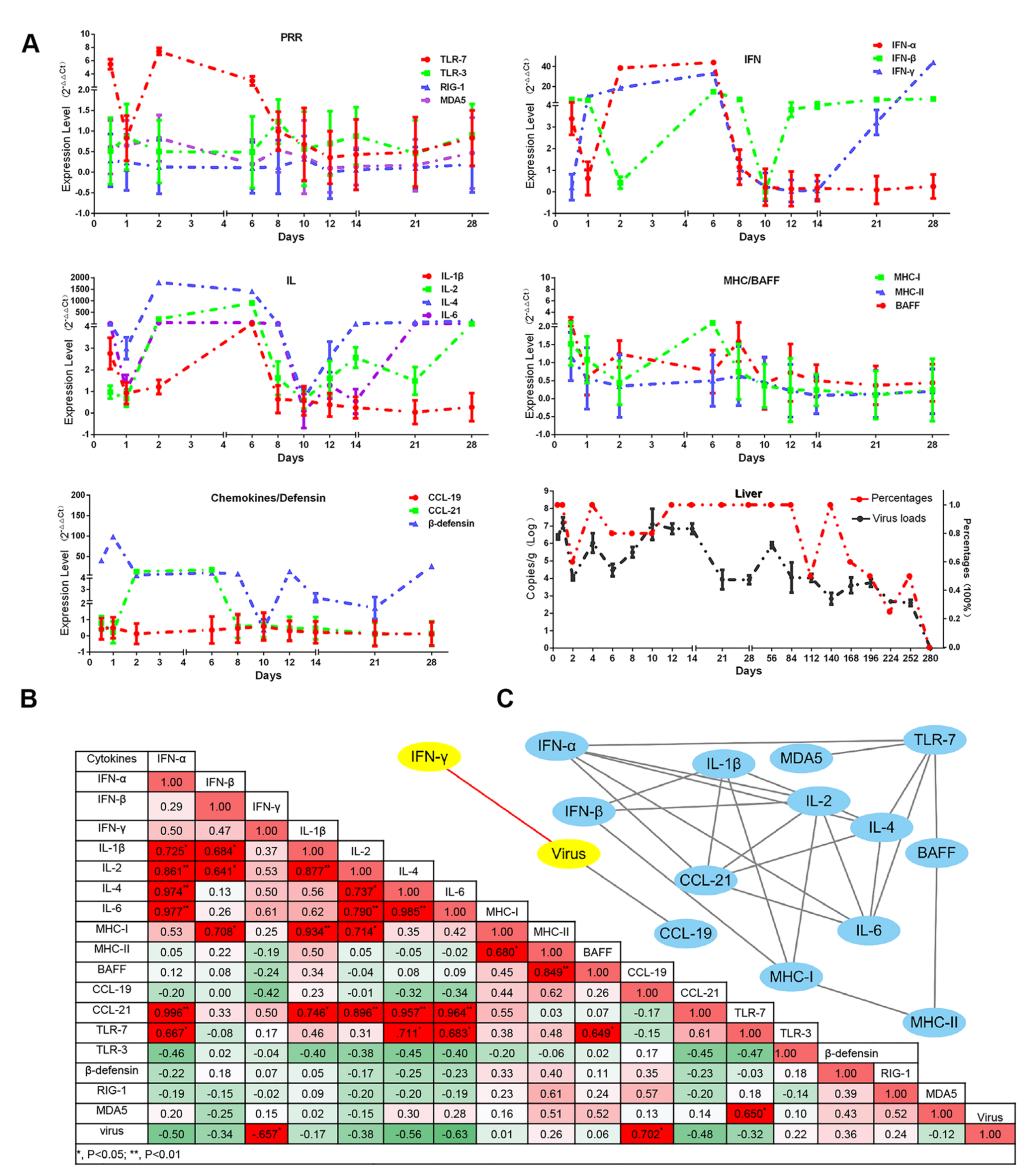
Dynamic immune-related gene expression profiles induced by DHAV-H strain and also the viral loads in liver **A.** Immune-related genes are displayed in each part according to their biological functions, such as the activation of innate immune responses (TLR7, TLR3, RIG-1 and MDA5), effective interferons (IFN-α/β/γ) and interleukins (IL-1β/2/4/6), chemokines (CCL19/21), MHC-I and MHC-II, β-defensin, and B cell activating factor (BAFF). The relative values for each immune related genes were calculated by 2^-ΔΔCt^ method by comparing with the non-infected group. The viral RNA copies (Log10/g) and their percentage in each group are displayed at the bottom right. Data are represented as mean +/- SD (*n* = 5). **B.** In order to understand the impact of virulence on immune networks, the correlations of each pair of immune related genes (*P* < 0.05 at least) were calculated using correlation analysis (Pearson). **C.** Those correlated pairs of immune related genes were visualized as immune network by Cytoscape software.

T-cell immunity is crucial for viral clearance and also humoral immunity. At early stage of infection, the numbers of CD4+ T -cells (Ths) and CD8+ T-cells (Tcs) significantly increased at 10dpi (Figure 3A, 3B, 3C). After that, both of them vanished from 28-dpi to 140-dpi and reoccurred at later stage of infection (Figure 3A, 3B, 3C). The numbers between Tcs and Ths at each interval were no significance (*P* > 0.05) (Figure 3D). In addition, immunohistochemistry assays indicated that both Tcs and helper T-cells Ths were located at the portal area of liver (Supplementary Figure 2, Supplementary Figure 3). In consistent with these observation, the dynamic changes in viral capsid load and viral RNA polymerases were also gradually increased and decreased during the later stage of infection (Supplementary Figure 4, Supplementary Figure 5) and also consistent with the decline of viral RNA copies (Figure 4A). These results indicated that recruitment of the T-cells in liver was associated with viral clearance. However, the comparatively lower CD4+ and CD8+ T-cells and persistent disappearance of them might lead to a chronic infection (40wk).

### Cytokine storms might be also associated with viral clearance

Humoral and cellular immunity are strongly modulated by immune-related cytokines. In this study, the interferons (IFN-α/β/γ) and interleukins (IL-1β/2/4/6) were highly up-regulated from 2-dpi to 6-dpi (Figure 4A). Interestingly, the period of cytokine storms was similar to that in kidney infected with DHAV-1 [33]. Simultaneously, viral copies in liver were decreased under the course of cytokine storms (Figure 4A). Subsequently, the cytokine storms were transiently quitted down at 10-dpi that might be caused by newly viral replications (Supplementary Figure 5). Consistently, the viral copies were significantly increased after the cytokine storms (Figure 4A). Additionally, Pearson correlation analysis indicated that the changes of interferons and interleukins were negatively correlated with the viral RNA copies, especially for IFN-γ (*P* < 0.05) (Figure 4B). In this study, TLR7 was highly upregulated from 2-dpi to 6-dpi, whereas other members of the Pattern Recognition Receptors (PRRs), TLR3, MDA5 and RIG-1, were slightly upregulated from 8-dpi to 14-dpi. Those observations indicated that TLR7 might be involved in the early cytokine storms and the other PRRs might be involved in the later cytokine expressions. Comparative analysis indicated that IL-4 and IFN-γ were the top two highly up-regulated cytokines from 12-dpi to 28-dpi (Supplementary Figure 6). While, the expression patterns of the Major Histocompatibility Complex (MHC) and chemokines were quite different. MHC-I was slightly upregulated from the time of infection to 6-dpi. CCL21 but not CCL19 was strongly elevated from 1-dpi to 6-dpi and disappeared from 8-dpi to 28-dpi. Additionally, BAFF, a member of the tumour necrosis factor (TNF) family, was temporarily upregulated at 8-dpi. The expression pattern of β-defensin was highly similar to that of interferons and interleukins (Figure 4A).

In order to understand their relationship, Pearson correlation analysis was used in this study. 27 pairs of genes were highly correlated and enriched in IFNs and interleukins (*P* < 0.05 at least) (Figure 4B). And we also identified that TLR7 was the top one PRRs correlated with both IFNs and interleukins (*P* < 0.05) (Figure 4C). Those results indicated that the activation of TLR7 pathway might be involved in early cytokine storms and viral clearance.

### Serious liver lesions in duck embryos was caused by infection of DHAV

**Figure 5:**
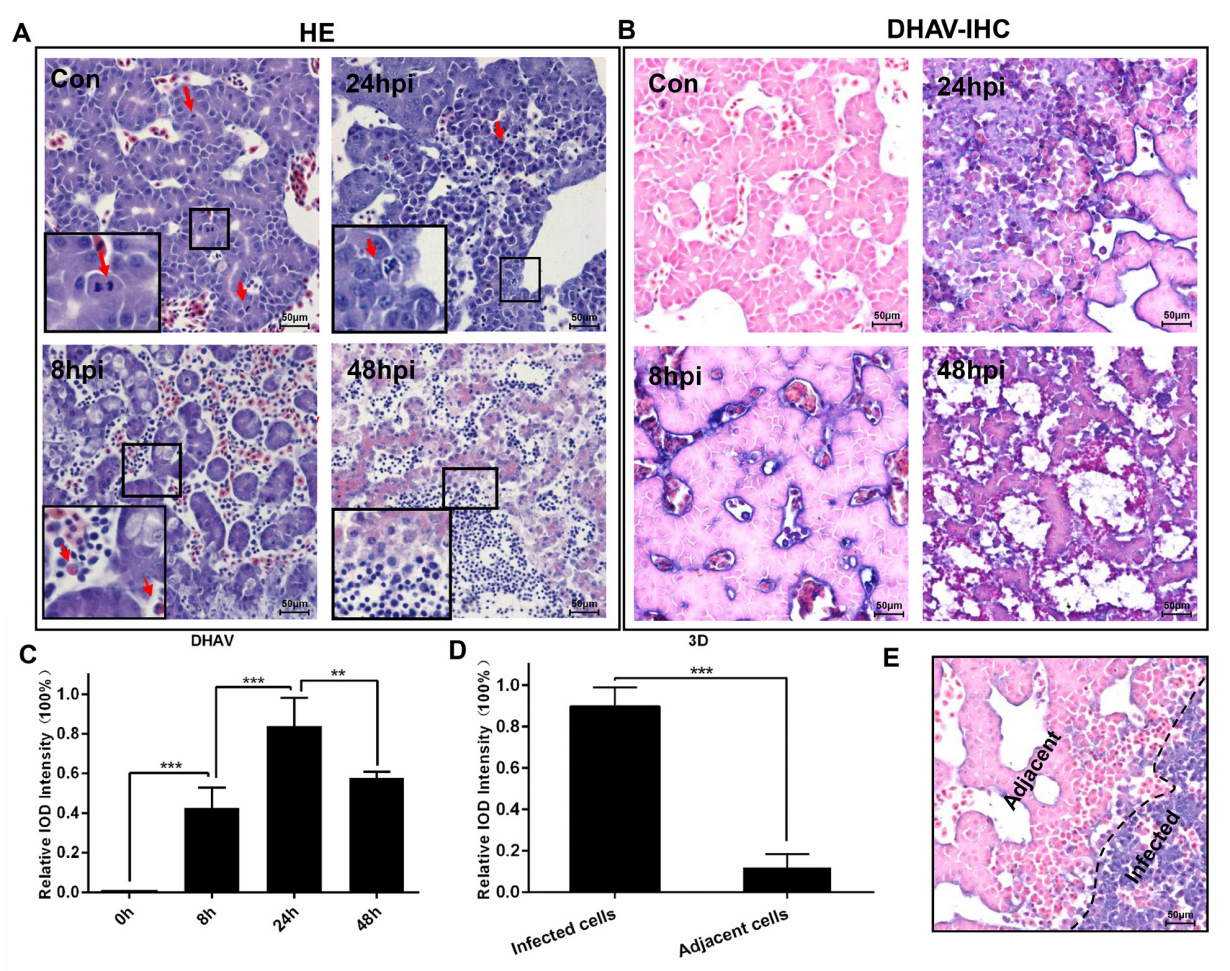
Microscopic lesions in the liver of duck embryos experimentally infected with DHAV-1 H strain **A.** Representative liver HE staining from 8-hpi to 48-hpi of DHAV is displayed (*n* = 3). Typical histopathological changes are exemplified at the corner of each photograph. (Con) represents pre-inoculation liver photomicrograph. **B.** Representative liver DHAV staining by IHC. **C.** The relative intensity of positive DHAV staining was calculated by Image-Pro Plus software. 100% Integral Optical Density (IOD ) was defined as the most positive staining in the course of infection. The significant levels of their expression were analysed by Student’s T-test, two way. **D.** The positive 3D^pro^ staining intensity of infected cells was significantly higher than the adjacent cells. **E**. Representative 3D protein staining at 24-hpi. The infected cells and adjacent cells were labelled. NS, No significance; *, *P* < 0.05; **, *P* < 0.01; ***, *P* < 0.001. The brightness and contrast are slightly modified to create a uniform background.

The duck embryos infected with DHAV progressed to liver lesion from apoptosis, cellular swelling to vacuolar degeneration (ballooning degeneration) and necrosis (Figure 5A). The normal liver cells had no histopathological change with many more mitotic cells. With prolonged time, then, the hepatocellular lesions were characterized by ballooning degeneration, and many more heterophilic granulocytes and neutrophile granulocytes were recruited to the liver at 8-hpi. However, they swelled, became round and even progressed to apoptosis after infection at 24-hpi. At the last stage of infection, all of the liver cells were characterized by necrosis, and plentiful neutrophil granulocytes were recruited to the liver. The histopathological changes were also accompanied with rapidly viral reproduction (Figure 5B, 5C). Of note, the expression levels of viral polymerase (3D protein) was apparently higher in those serious infected cells than adjacent cells (Figure 5D, 5E).

### Activation of primary duck embryo HSCs was associated with hepatocellular lesions

**Figure 6:**
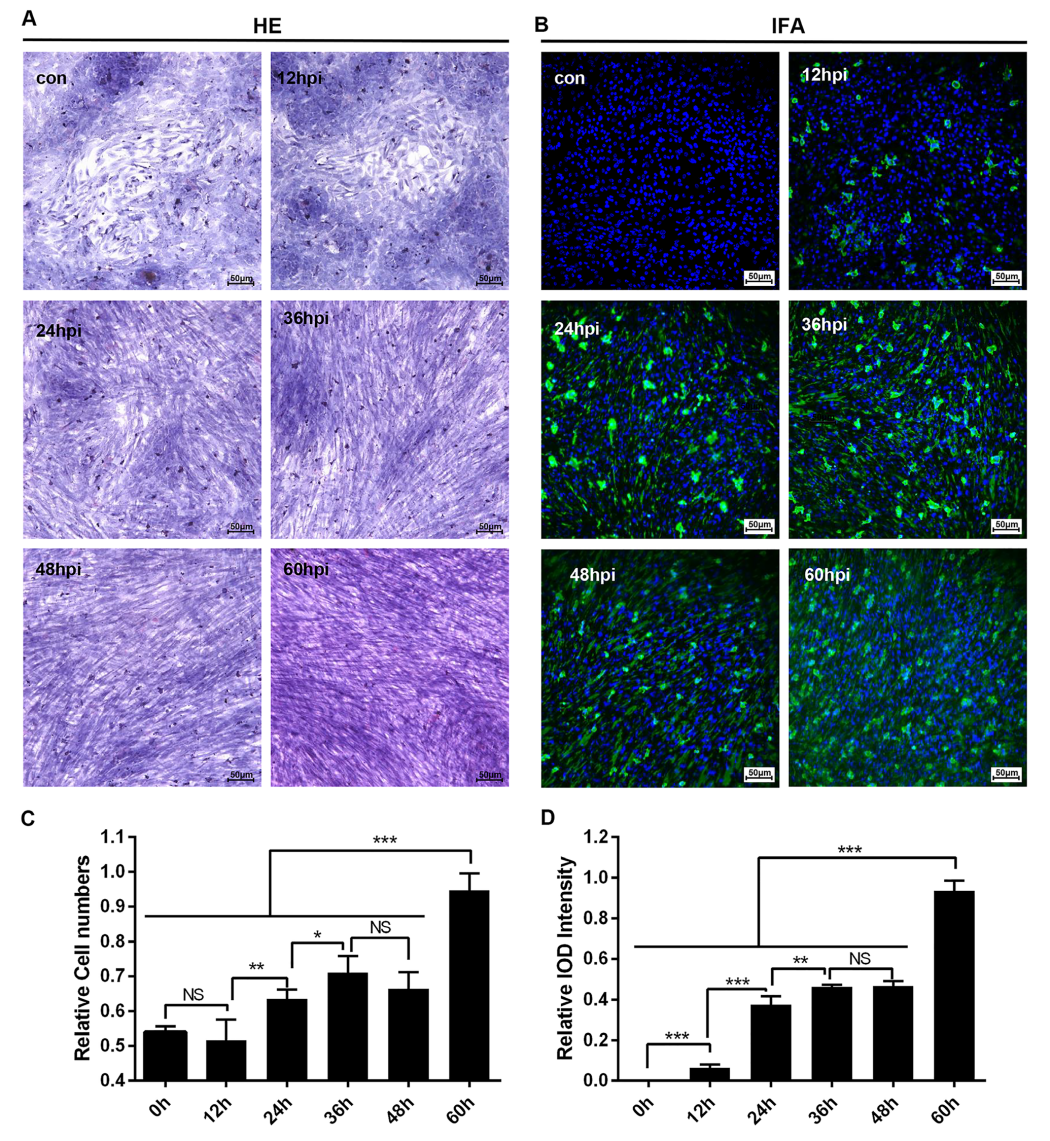
Microscopic lesions and virus replication of duck embryo HSCs infected with DHAV-1 H strain **A.** Representative duck embryo HSC HE staining (*n* = 2). **B.** Virus detection through indirect immunofluorescence after DHAV infection from 12-hpi to 60-hpi are displayed on the left and right (n = 2). **C.** The relative cell numbers were calculated by Image-Pro Plus software. **D.**The intensity of positive staining were also calculated by Image-Pro Plus software. The significant levels of their expression or numbers were analysed by Student’s T-test. NS, No significance; *, *P* < 0.05; **, *P* < 0.01; ***, *P* < 0.001. The relative cell numbers was calculated by comparing the slides with maximum cells (1.0). The 100% Integral Optical Density (IOD) intensity was defined as the slides with the most positive staining. The brightness and contrast are slightly modified to create a uniform background.

Cultured cells used for certain virus infections provided the potential for researchers to address the pathogenesis caused by the virus *in vivo*. Fortunately, primary duck embryo liver cells were available for DHAV infection. Meanwhile, infection of primary duck embryo liver cells could also induce activation of HSCs. As is shown in Figure 6, morphogenesis of quiet HSCs was significantly transformed to an activated type after hepatocellular lesion caused by DHAV. This process was associated with virus infection such that virions were adsorbed at the cell membrane at 12-hpi and then produced much more progeny virus in the cytoplasm from 36-hpi to 60-hpi. Additionally, virus replication was also accompanied by serious cytopathic changes characterized by activation of HSCs and disappeared hepatocellular islands (Figure 6A, 6B). It is interesting that activated HSCs numbers and viral replication were both gradually increased (Figure 6C, 6D), which may be involved in liver repair when undergo the infection of DHAV.

### Phylogenetic analysis of DHAV and HAV genomes and fibrosis-related genes in humans and ducks

**Figure 7:**
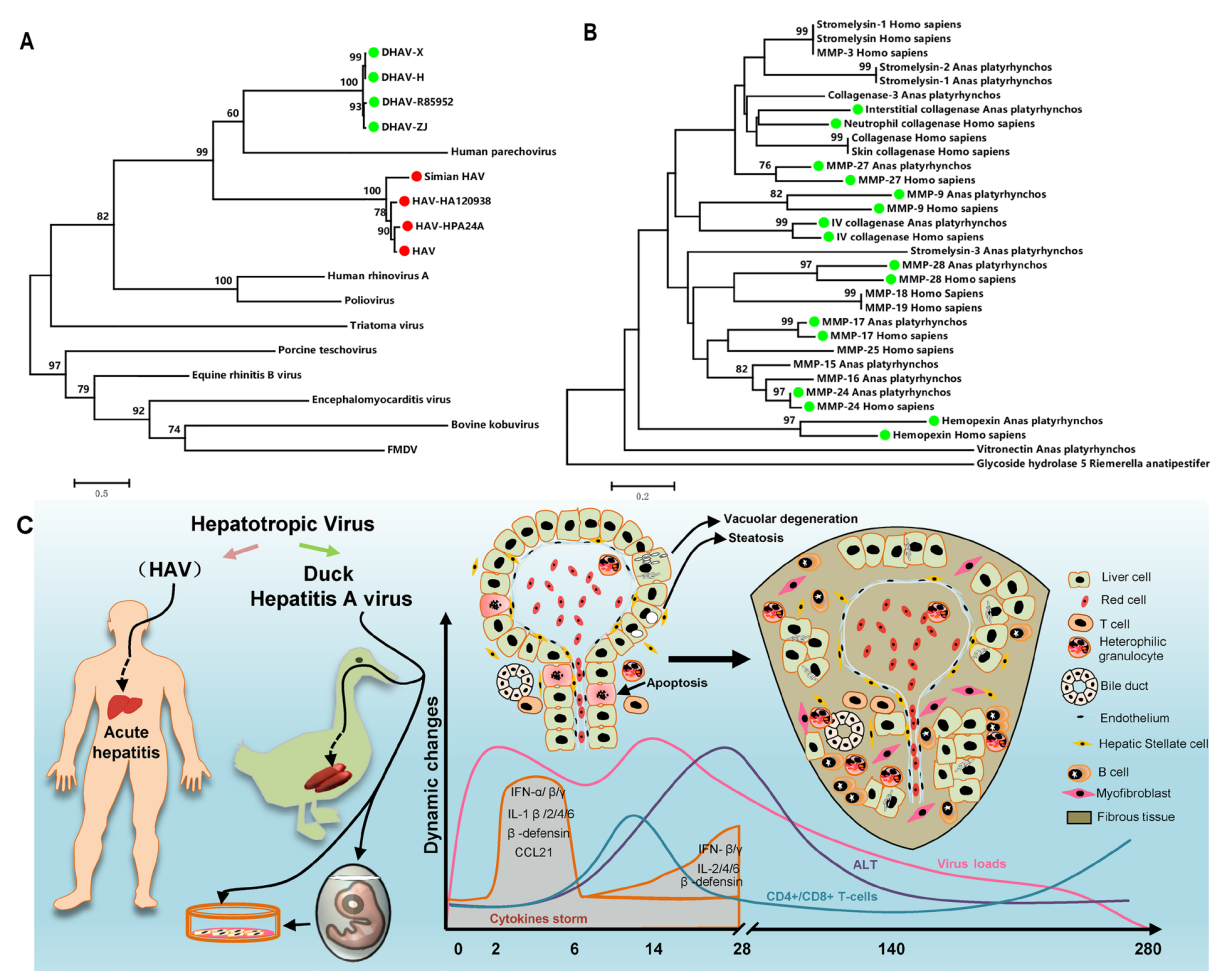
Comparatively phylogenetic relationship with “Hepatitis A virus” and fibrosis-related genes in humans and ducks and the process of liver injury and repair during DHAV infection **A.** The phylogenetic tree of the DHAV-related genes was constructed from the complete genomic sequences of the picornaviruses using the neighbour-joining method. **B.** The complete amino acid sequences of the fibrosis-related genes in the genomes of humans and ducks were used to construct a phylogenetic tree using the neighbour-joining method. The percentage of replicate trees in which the associated taxa clustered together in the bootstrap test (1000 replicates) are shown above the branches. **C.** The duck has a similar liver disease caused by avian hepatropic virus (DHAV). This virus can also be cultured in duck embryos or its primary cells. During the period of infection, virus rapidly replicates to a high level, and then the transient cytokine storm reduces the virus to a comparatively lower level and gradually disappears until 280dpi. Hepatocellular injury was characterized as vacuolar degeneration, steatosis and apoptosis at early stage of infection. While those injured cells were replaced by collagenous fiber at later stage of infection. Those fibres may be secreted by myofibroblast when hepatic stellate cells were activated by injured liver cells. Meanwhile, strong recruitment of plasmocytes and heterophilic granulocytes were identified by HE assays. However, the comparatively lower CD4+ and CD8+ T-cells recruited to liver might lead to a persistent infection. Those immune cells may combine with interferons and interleukins to eradicate viral replication from liver.

To identify their sequence similarity, multiple alignments of DHAV and HAV were used for this purpose (https://blast.ncbi.nlm.nih.gov/Blast.cgi). They shared 50% nucleotide sequence identity with 20% protein sequence identity. Phylogenetic analysis provided curial information to understand the genetic relationship between different genera. In this analysis, though DHAV was related more closely to the genus *Parechovirus*, the avian hepatotropic virus, *Avihepatovius*, was also very close to the human Hepatitis A virus [34] (Figure 7A). Therefore, DHAV should be recognized as an HAV-related animal virus in birds.

To find the potential of ducks as an animal model for liver fibrosis, fibrosis-related genes in the genomes of humans and ducks were used to construct a phylogenetic tree. It has been shown that approximately 50% (16/32) of fibrosis-related genes had high homology between humans and ducks (Figure 7B). This result indicated that the mechanism of fibrogenesis might be similar in mammal and avian species, at least in this study.

## DISCUSSION

Non-primate hepatitis animal models would be beneficial due to the lower cost to maintain, the availability to house within laboratories, and especially for the great concern about the shortage of primate models and phase out of use of them in research. Optional rodent models for hepatotropic virus research are necessary. Unfortunately, the currently developed humanized mouse models have limited life cycles or no viral replication [35]. The most recently discovered horse model provided an alternative large animal model for chronicity studies over several years. However, their higher expense and the shortage of immunological and biochemical reagents were the major obstacles faced by researchers [27]. Here, we provide an alternative small animal model that remedied irreparable defects in the HBV-related duck model owing to limited cytopathic effect. Fortunately, mature ducks infected with DHAV can induce acute and chronic hepatitis, which can be used to mimic the process of liver injury in the long term. Those advantages include the ability to perform acute hepatitis and chronic studies for fibrosis genesis, cirrhosis and hepatocellular regeneration and enough mass for serial liver biopsies.

In this longitudinal study, experimental inoculation of mature ducks resulted in a course of liver lesions, repair, and regeneration similar to HAV in primates, especially in the early stage of infection [20, 36]. During the early stage of infection, liver injury was characterized by vacuolation, acidophilic degeneration, steatosis and infiltration of neutrophil granulocytes. Coincident with liver injury, the serum biomarkers, ALT and AST, were significantly increased (*P* < 0.05). Similarly, human acute viral hepatitis also resulted in hepatocellular acidophilic degeneration and recruitment of a plentiful of lymphocytes [17]. The histological lesions in ducks associated with acute viral hepatitis were very similar to that in EHCV and HAV infection [20, 27]. Viral RNAs can be sensed by cytosolic PRRs, including Toll-like receptor 7 (TLR7), TLR3, retinoic acid-inducible gene I (RIG-I) and melanoma differentiation-associated gene 5 (MDA5) [37]. Activation of both types of PRRs by ssRNA or dsRNA was subjected to induce type I interferon and other inflammatory cytokines [38, 39]. However, except for TLR7, the other three members of the PRRs were only slightly up-regulated at 8-dpi to 14-dpi. This finding was supported by that the TLR3 signalling pathway was also disrupted through the cleavage of TRIF directed by a 3CD intermediate [40]. HAV infection also suppresses the production of Type I IFNs through the RIG-I/MDA5 pathway, through which signal transduction is blocked through the cleaving activity of 3ABC protease to Mitochondrial Antiviral Signalling Protein (MAVS) [40, 41]. The infiltration of neutrophil granulocytes was accompanied by strong elevation of CCL21 but not CCL19. This result was consistent with larger and more organized infiltrates directed by mouse ectopic expression of CCL21 [42]. Expression of CCL21 was also involved in the organization of inflammatory lymphoid tissue and the recruitment of T-cells, which may promote fibrogenesis via activation of chemokine receptor 7 (CCR7) on HSCs during chronic hepatitis C [43]. Previous studies indicated that recruitment of Tc and Th cells to the liver could increase the efficiency of viral clearance [44, 45]. In this study, however, the comparatively lower T-cell response may lead to a chronic DHAV infection.

During the later stage of infection, liver lesions were characterized by serious vacuolation, steatosis, interface hepatitis and progressive fibrosis. Many T-cells, plasm cells and heterophilic granulocytes were recruited to the liver to resist viral infection until the last stage of virus clearance. During the sustained battle between virus and host, the injured liver cells were replaced either by fibrosis or regenerated parenchyma cells. Our data indicated that the time of sustained infection in the liver (40 wk) was very similar to that of HAV-infected chimpanzees (35 to >48 wk) and much longer than HCV infection (10-20 wk) [20]. The sustained expression of viral RNA polymerases reflects ongoing viral replication (Supplementary Figure 5), which was consistent with viral RNA copies (Figure 2). Combined with these evidences, the ducks infected DHAV could progress to chronic viral hepatitis. Interestingly, viral RNA copies peaked at 2 wk, dramatically declined at 4 wk, and then gradually vanished until 280-dpi in liver. The dynamic changes were negatively related to the intensity of Tc responses, interferons and interleukins and positively correlated with liver injury. To further investigate the role of cytokines in fibrogenesis, conjoint analysis indicated that Th1/Th2 cytokines (IFN-γ/IL-4) were inclined to Th2 immune responses from 14-dpi to 28-dpi, which was consistent with the progress of fibrosis (Supplementary Figure 1, Supplementary Figure 6). These results indicated that the IL-4 might be the predominantly profibrogenic cytokines, which is consistent with CCl_4_-induced fibrosis in mice [12]. Those characteristics of liver injury, fibrosis and regeneration should be recognized as chronic viral hepatitis. To understand the potential of Peking ducks as a small model for research on human acute and chronic hepatitis, phylogenetic analysis of the homology of avian hepatotropic virus (DHAV) and human hepatovirus indicated that although DHAV was close to the genus *Parechovirus*, it was also highly related to HAV in the family *Picornaviridae* [34]. Additionally, phylogenetic analysis of ECM deposition-related genes in ducks and humans suggested that half of them shared higher homology (Figure 7B). Those data indicated that DHAV, the HAV-related avian hepatotropic virus, could induce very similar liver injury in human caused by HBV or HCV. This small model may benefit the pathogenesis and development of therapeutic strategies for liver injury in humans [10, 46].

Additionally, DHAV could be cultured in both duck embryos and primary duck embryo liver cells, which provided much more availability to address pathogenesis *in vivo* and *in vitro*. Serious liver injury caused by DHAV in duck embryos was characterized by apoptosis, cellular swelling to vacuolar degeneration (ballooning degeneration), apoptosis or necrosis, which was mainly caused by viral non-structural proteins, such as 2A^pro^ and 3C^pro^ or their precursors [40, 41, 47]. In addition, duck embryo HSCs could be stimulated and activated by DHAV infection (Figure 6). The activation of HSCs is involved in differentiation and proliferation of fibroblasts. Our data indicated that DHAV can be widely cultured in duck embryos and primary liver cells and can infect mature ducks progressing from acute hepatitis to chronic hepatitis.

In summary, the avian hepatotropic virus (DHAV) can induce apparently liver injury in both mature ducks and their embryos. Due to similar phenotype of liver injury, the pathogenesis of avian hepatotropic virus might be similar to some of the human hepatotropic virus. This small animal model provides the potential to address the process of acute and chronic viral hepatitis, fibrosis and hepatocellular regeneration, in a manner that was economical and easy to acquire and house in the laboratory (Figure 7C). Comparative and correlation studies between the viral life cycle and liver injury and repair are likely to provide insight into the mechanisms of viral persistence or clearance in chronic HBV or HCV infection.

## MATERIALS AND METHODS

### Ethics statement

The animal study was also performed in strict accordance with the recommendations in the ARRIVE guidelines (http://www.nc3rs.org.uk/arrive-guidelines). The animal experiment has been approved by the committee of experiment operational guidelines and animal welfare of Sichuan Agricultural University, China (the approved permit number is XF2014-18). All ducks were handled in compliance with the animal welfare regulations and maintained according to standard protocols.

### Virus and primary duck embryo liver cell preparation

The DHAV-H strain (GenBank: JQ301467.1) was propagated in 9- to 11-day-old duck embryos by standard procedures. The embryos that died at 36-72 hpi were harvested. The homogenate of the allantoic fluid of duck embryos was stored at -80°C until use. The virus, at a concentration of 4.56 × 10^8^ copies/ml as determined by quantitative real-time PCR (qPCR), was used to infect ducks [48].

Duck embryo liver cells were prepared using 9- to 11-day-old duck embryonated eggs according to definitions within the program. The dispersed cells were dispensed into flat-bottomed, 12-well culture plastic plates (Corning Inc., New York, USA) with cover slips. The cells were cultured in Dulbecco’s modified Eagle’s medium with 10 % foetal bovine serum. Then, the cells were maintained in another medium containing 3 % foetal bovine serum. Cells were maintained at 37^°^C with 5 % CO_2_.

### Experimental design

One hundred and five female ducks were randomly divided into 21 groups. Group 1 to group 20 received 1 ml of DHAV-H strain (Genebank: JQ301467) (4.56×10^8^ copies/ml) by intramuscular injection, and group 21 was injected with an equal volume of 0.85% physiological saline as a negative control. The livers of 20 experimental groups were collected according to the time post-infection, including 1/2d, 1d, 2d, 4d, 6d, 8d, 10d, 12d, 14d, 21d, 28d, 56d, 84d, 112d, 140d, 168d, 196d, 224d, 252d, and 280d. One hundred milligrams of liver specimens was weighed and then immediately cryopreserved in liquid nitrogen until processing for RNA isolation. The adjacent tissues were used for histopathological examination and immunohistochemistry.

To identify the hepatocellular lesions caused by DHAV, duck embryos were also used to address this question. Twelve 6-day-old duck embryos were randomly divided into 4 groups. Group 1 to group 3 received 0.2 ml of virus (4.56×10^8^ copies/ml) at the allantoic cavity, and group 4 was injected with an equal volume of 0.85% physiological saline as a negative control.

The duck embryo liver cells were cultured in the 12-well culture plastic plates divided into 6 groups. Group 2 to group 6 received 0.1 ml of virus (4.56×10^8^ copies/ml), and group 1 received an equal volume of 0.85% physiological saline as a negative control.

### qPCR

Total cellular RNA was isolated from 100 mg of liver tissue using the RNAiso plus Reagent (TaKaRa, Japan) according to the manufacturer’s protocols. The RNA isolated from each specimen needed to detect immune-related genes was used to carry out reverse transcription to produce cDNA with the PrimeScriptTM RT Reagent Kit according to the manufacturer’s instructions (TaKaRa, Janpan). Viral copies were detected by previously established methods in our laboratory [48]. Seventeen immune-related genes (IL-1β, IL-2, IL-4, IL-6, IFN-α, IFN-β, IFN-γ, MHC-I, MHC-II, CCL19, CCL-21, BAFF, TLR3, TLR7, β-defensin, RIG-1 and MDA5) and a housekeeping gene (glyceraldehyde-3-phosphate dehydrogenase (GAPDH)) were also detected according to the previously established protocols [33].

### HE and Masson staining

After administering sodium pentobarbital anaesthesia, the livers from the same samples used for transcriptional analysis were fixed in 4% paraformaldehyde, dehydrated, embedded in paraffin, sectioned into 4-μm-thick sections and stained with haematoxylin and eosin (HE) and Masson using standard procedures. Additionally, the duck embryos and duck embryo liver cells infected with the DHAV-H strain were fixed in 4% paraformaldehyde first, and then their liver tissues were also used for HE staining using standard procedures.

### Flow cytometry analysis

To validate the identity of CD4+ and CD8a+ positive staining, the liver tissues used for IHC assays were also detected by flow cytometry (BD FACS Verse™, San Jose, CA, USA). Uninfected liver tissues were used as a negative control. Briefly, the positive cells were incubated with mouse anti-duck CD4 or CD8а monoclonal antibody (8μg/ml) followed by FITC-coupled goat anti mouse secondary antibody solutions (1:500 dilution) (Beyotime Biotechnology). Then, the percentage of FITC-positive cells were determined by flow cytometry.

### I**mmunohistochemistry**

Paraffin-embedded liver tissues were deparaffinized in xylene and rehydrated in graded alcohols. Briefly, the standard protocol were previously established for detecting viral capsid and 3D polymerase and also double IHC staining for detecting viral capsid and CD4+ or CD8+ T cells.[33].

### Indirect immunofluorescence

Following the 12 h, 24 h, 36 h, 48 h and 60 h incubation at 37°C after infection of the DHAV-H strain (100 μL), the cells were washed with phosphate buffered saline solution (PBS; 0.15 mol/L, pH 7.2) and fixed for 30 min at room temperature (RT) with 4% paraformaldehyde. After fixation, the paraformaldehyde was removed and the cells were permeabilized with 0.5% Triton X-100 for 10 min at RT and incubated in blocking buffer (PBS, 5% BSA) for 30 min at RT. The primary rabbit anti DHAV polyclonal antibody was diluted in 0.5% BSA and incubated with cells for 1 h at RT. After being washed with PBS, the coverslips were incubated with goat anti-rabbit Alexa Fluor^®^ 488 conjugate (Life technologies, USA) diluted in 0.5% BSA for 1 h at RT. After counterstaining, coverslips were washed three times with PBS, mounted with fluorescence mounting media (KPL, Gaithersburg, MD), and examined with a Nikon Microscope. We used cells infected with PBS as a negative control.

### Phylogenetic analysis of DHAV and HAV and the fibrosis-related genes in humans and ducks

The DHAV-related genus in the family of *Picornavirdae* was used to identify the neighbour genus. The sequences of fibrosis-related genes in human and duck were also used to compare their evolutionary relationship. The phylogenetic tree was constructed by Mega 6.0 with the neighbour-joining (NJ) method and 1000 bootstrap analysis [49]. The members of picornavirus and fibrosis-related genes in ducks and humans used for phylogenetic analysis are listed in Supplementary Table 3.

### Statistical analyses

Relative gene expression data were analyzed using the 2^-ΔΔCt^ method by comparing with the control group injected with 1 ml normal saline (NS) [50], and ΔCt values were determined by subtracting average Ct values of the endogenous control gene GAPDH from average Ct values of target genes. The photographs were generated using GraphPad Prism 5 software. In order to understand the impact of virulence on immune networks, the correlations of each pair of immune related genes were calculated using correlation analysis (Pearson) using SPSS software. Those correlated pairs of immune related genes were visualized as immune network by Cytoscape software. The significant levels in this study determined using Student’s t test (Two-tail, α=0.05).

## SUPPLEMENTARY MATERIALS FIGURES AND TABLES



## Abbreviations

HAV: Hepatitis A virus
DHAV: Duck Hepatitis A virus
HBV: Hepatitis B virus
HCV: Hepatitis C virus
HEV: Hepatitis E virus
qPCR: quantitative real-time PCR
Tc: cytotoxic T cell
Th: helper T-cell
dpi: day(s) post infection
