# Supplementary material for: The neglected avian hepatotropic virus induces acute and chronic hepatitis in ducks: an alternative model for hepatology
Source: Oncotarget. 2017 Jul 5;8(47):81838–51. doi: 10.18632/oncotarget.19003 (PMC5669852; doi:10.18632/oncotarget.19003)
Supplement: Supplementary file 1 [file oncotarget-08-81838-s001.pdf]

The neglected avian Hepatovirus induces acute and chronic hepatitis in mature ducks: an excellent model for hepatology

SUPPLEMENTARY MATERIALS

Table of contents

Supplementary materials and methods…………………………………………….…………..….2

Supplementary Figure legends…...……………………………………………………..…....7

Fold changes of immune related genes induced by DHAV-H strain………………………9

Dynamic changes of virus load in liver…………………………………………………..……10

Members of picornavirus and fibrosis related genes in ducks and humans………..…11

Primer sequences used in this study…………………….………………………………………12

References ……..…………………………………………………………………….………………13

Supplementary materials and methods

Ethics statement

The 160-day-old female Peking ducks were purchased from Mianying company (http://www.mianying.com), and this study was performed in strict accordance with the recommendations in the ARRIVE guidelines. The animal study was also performed in strict accordance with the recommendations in the ARRIVE guidelines (http://www.nc3rs.org.uk/arrive-guidelines). The animal experiment has been approved by the committee of experiment operational guidelines and animal welfare of Sichuan Agricultural University, China (the approved permit number is XF2014-18). All ducks were handled in compliance with the animal welfare regulations and maintained according to standard protocols. All surgeries were performed on animals under sodium pentobarbital anaesthesia, and all efforts were made to minimize suffering.

Viral strains

The DHAV-H strain (GenBank: JQ301467.1) was propagated in 9- to 11-day-old duck embryos by standard procedures. The embryos that died at 36-72 hpi were harvested. The homogenate of the allantoic fluid of duck embryos was stored at -80°C until use. The virus, at a concentration of 4.56×10^8 copies/ml as determined by quantitative real-time PCR (qPCR), was used to infect ducks.

RNA isolation and cDNA preparation

Total cellular RNA was isolated from 100 mg of liver using the RNAiso plus Reagent (TaKaRa, Japan) according to the manufacturer's protocols. Briefly, 100 mg of each tissue was finely powdered in a ceramic mortar with a sufficient amount of liquid nitrogen, followed by addition of 1000 μl of RNAiso Reagent and then delamination using 200 μL of chloroform with intense shaking for 15 s. The nucleic acids were suspended by centrifugation (12,000 × g at 4°C for 15 min), and then 500 μL of isopropanol was added. The RNA was then precipitated by centrifugation (12,000 × g at 4°C for 15 min) and washed with 75% ethanol. After air drying, the RNA pellet was resuspended in 50 μL of

diethyl pyrocarbonate (DEPC)-treated water and stored at −70°C until use. The total RNA was detected spectrophotometrically using the Smartspec-3000 (Bio-Rad, USA) and agarose gel electrophoresis to confirm the quality.

RNA isolated from each specimen needed to detect immune-related genes was treated with 1.0 μL of gDNA Eraser (Perfect Real Time, Janpan) for two min at 42°C to remove the potential contaminating genomic DNA (gDNA) and then used to carry out a reverse transcription to produce cDNA with the PrimeScriptTM RT reagent Kit according to the manufacturer's instructions (TaKaRa, Janpan).

Determination of primer/probe efficiency and experimental standard for TaqMan qPCR

We amplified the 440-bp PCR products from DHAV-H strains using a pair of outer primers (forward primer 5'-acaatgacccagccttag-3', reverse primer 5'-ccactgtatcttcccttc-3') designed according to the conserved region in the 3D gene (GenBank: EF064886) of DHAV-1 (Invitrogen, USA) [1]. The purified 440-bp DNA fragment was cloned into the pGEM-T vector (Promega, USA) according to the manufacturer's instructions. The reverse sequence of the insert was confirmed, and the plasmid, linearized by the Sal I restriction enzyme, was subjected to in vitro transcription using the TranscriptAid T7 High Yield Transcription Kit (Thermo Fisher Scientific, USA). The transcribed RNA was treated with DNase I (Thermo Fisher Scientific) to remove linearized plasmid and extracted using RNAiso plus Reagent (TaKaRa, Janpan). The purified RNA templates were quantified spectrophotometrically using the Smartspec-3000 (Bio-Rad). Ten-fold serial dilutions of the RNA template (1.46×10^8–1.46×10^3 copies/μl) with a pair of inner primers (forward primer 5'-tgatgagatatggcaggtagaagga-3', reverse primer 5'-cacgcaagctgattcacaataga-3') and TaqMan probe (5'-FAM-tgtgttcaggatccccatgtactaccgtg-TAMRA-3') were used to establish the standard curve [1].

To simplify the amplification process and minimize the risk of contamination, one-step qPCR based on TaqMan technology was performed using the One Step PrimeScript RT-PCR Kit (Takara) according to the manufacturer's instructions. Agarose gel electrophoresis and Blast primer

(http://www.ncbi.nlm.gov/BLAST/) analyses were utilized to verify the specific product. We used conditions optimized in our previous study to establish the standard curve and detect the viral copy numbers in specimens using the iCycler iQ Multicolor Real-Time PCR Detection System (Bio-Rad) [1]. A regression curve was constructed by plotting the cycle threshold (Ct) values versus the logarithm of the RNA copy numbers.

qPCR

Seventeen immune-related genes (IL-1β, IL-2, IL-4, IL-6, IFN-α, IFN-β, IFN-γ, MHC-I, MHC-II, CCL19, CCL-21, BAFF, TLR3, TLR7, β-defensin, RIG-1 and MDA5) and a housekeeping gene glyceraldehyde-3-phosphate dehydrogenase (GAPDH) were detected by qPCR. Primer sequences for detecting the immune gene transcripts were previously published [2] (Table S4). The primer sequences for IL-4, BAFF, CCL19, CCL21, TLR3, β-defensin, RIG-1 and MDA5 were newly designed in this study using Primer 3 input version 0.4.0 (http://bioinfo.ut.ee/primer3-0.4.0/), and non-specific products were eliminated by using online NCBI BLAST searches. All primers used in this study are shown in Table S4. Expression levels of mRNA transcripts were determined by qPCR using the SYBR®Premix Ex Taq™ II (Tli RNaseH Plus) Kit (Takara). The amplification procedure was performed in a 20 μL reaction volume containing 8 μM of each primer and 2 μl of cDNA. The following thermal cycling conditions were used: PCR initial activation at 95°C for 30 s, 45 cycles of denaturation at 95°C at 5 s, annealing and extension at 58.2°C for 30 s.

HE and Masson staining

After administering sodium pentobarbital anaesthesia, the livers from the same samples used for transcriptional analysis were fixed in 4% paraformaldehyde, dehydrated, embedded in paraffin, sectioned into 4-μm-thick sections and stained with haematoxylin and eosin (HE) and Masson using standard procedures. Additionally, the duck embryos and duck embryo liver cells infected with the DHAV-H strain were fixed in 4% paraformaldehyde first, and then their liver tissues were also used for HE staining using standard procedures.

Immunohistochemistry

Paraffin-embedded liver tissues were deparaffinized in xylene and rehydrated in graded alcohols. For antigen retrieval, slides were boiled in Tris/EDTA PH 9.0 for 20 min. Next, 0.01 M HCl was used to block endogenous alkaline phosphatase for 15 min at room temperature. The slides were incubated in 5% BSA blocking solution followed by overnight incubation at 4℃ in rabbit anti DHAV polyclonal antibody (1:20 dilution) and rabbit anti RNA polymerases (3D) polyclonal antibody (1:20 dilution), respectively. Then, alkaline phosphatase-conjugated goat anti rabbit IgG (1:1000 dilution, Life Technology) was incubated for 30 min at 37℃. The positive staining was coloured with BCIP/NBT solution for 20 min at room temperature and counterstained with nuclear fast red for 20 min at room temperature (RT). Additionally, for double staining of DHAV and CD4+ or CD8α+positive T-cells, samples were first incubated with a cocktail of rabbit anti DHAV polyclonal antibody and mouse anti duck CD4 or CD8a monoclonal antibody (1:20 dilution and 1:200 or 1:100 dilution, respectively), followed by the mixture of HRP- or alkaline phosphatase-coupled goat anti rabbit or donkey anti mouse secondary antibody mixture. Then, positive staining was coloured with DAB solution for 10 min and permanent red solution for 15 min at RT and counterstained with haematoxylin.

Phylogenetic analysis of DHAV and HAV and the fibrosis-related genes in humans and ducks

The DHAV-related genus in the family of Picornavirdae was used to identify the neighbour genus. The sequences of fibrosis-related genes in human and duck were also used to compare their evolutionary relationship. The phylogenetic tree was constructed by Mega 6.0 with the neighbour-joining (NJ) method and 1000 bootstrap analysis [3]. The members of picornavirus and fibrosis-related genes in ducks and humans used for phylogenetic analysis are listed in Table S3.

Real-time PCR and statistical analyses

Relative gene expression data were analyzed using the $2^{-\Delta\Delta Ct}$ method by comparing with the control group injected with 1 ml normal saline (NS) [4], and ΔCt values were determined by

subtracting average Ct values of the endogenous control gene GAPDH from average Ct values of target genes. The photographs were generated using GraphPad Prism 5 software. In order to understand the impact of virulence on immune networks, the correlations of each pair of immune related genes were calculated using correlation analysis (Pearson) using SPSS software. Those correlated pairs of immune related genes were visualized as immune network by Cytoscape software. The significant levels in this study determined using Student's t test (Two-tail, α=0.05).

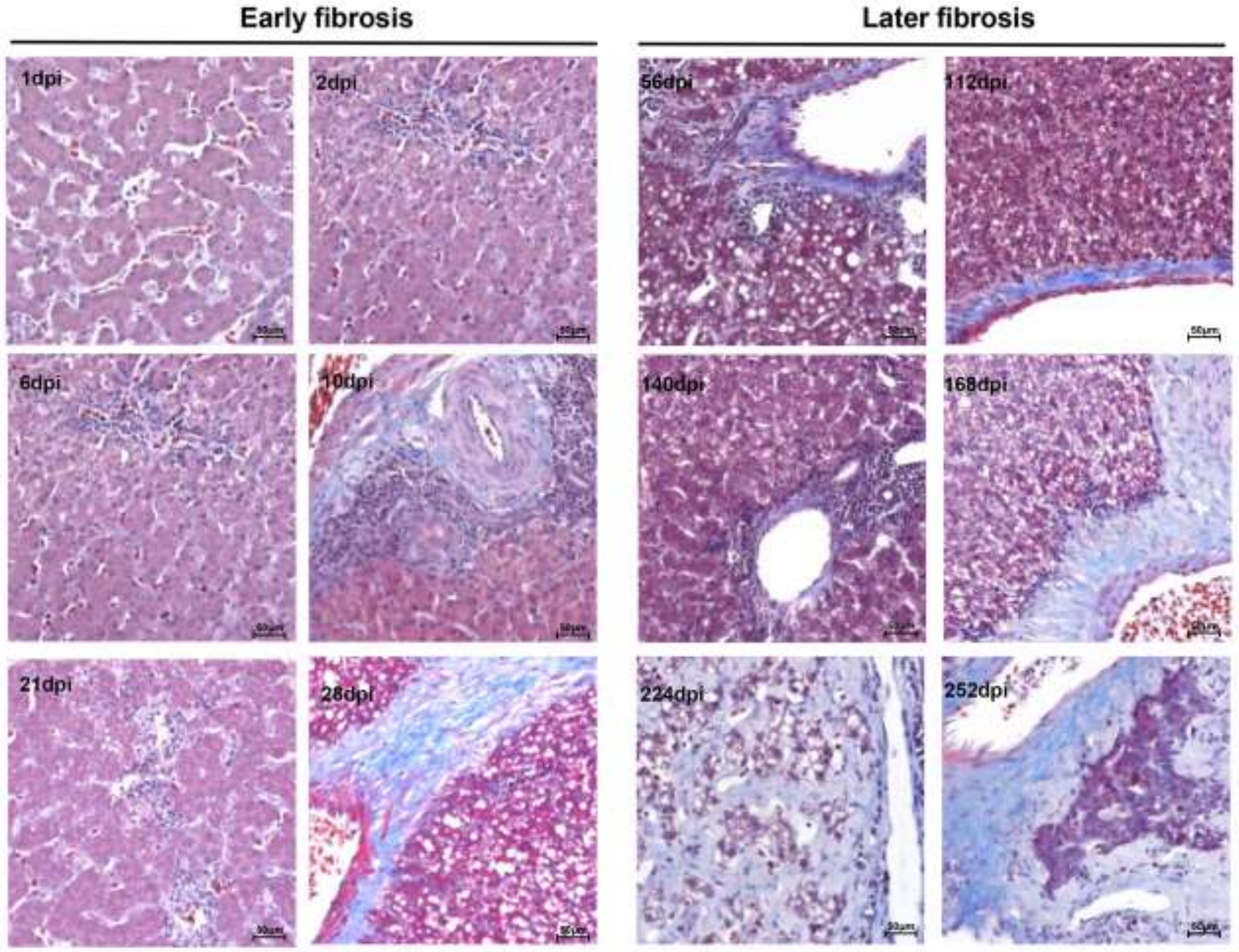

Supplementary Figure 1: Liver fibrogenesis in mature ducks experimentally infected with DHAV-1 H strain. The representative liver Masson staining from 1d to 280d post-infection with DHAV are also displayed with two stages, early infection and later infection. Blue colour represents collagenous fibres. The brightness and contrast are slightly modified to create a uniform background.

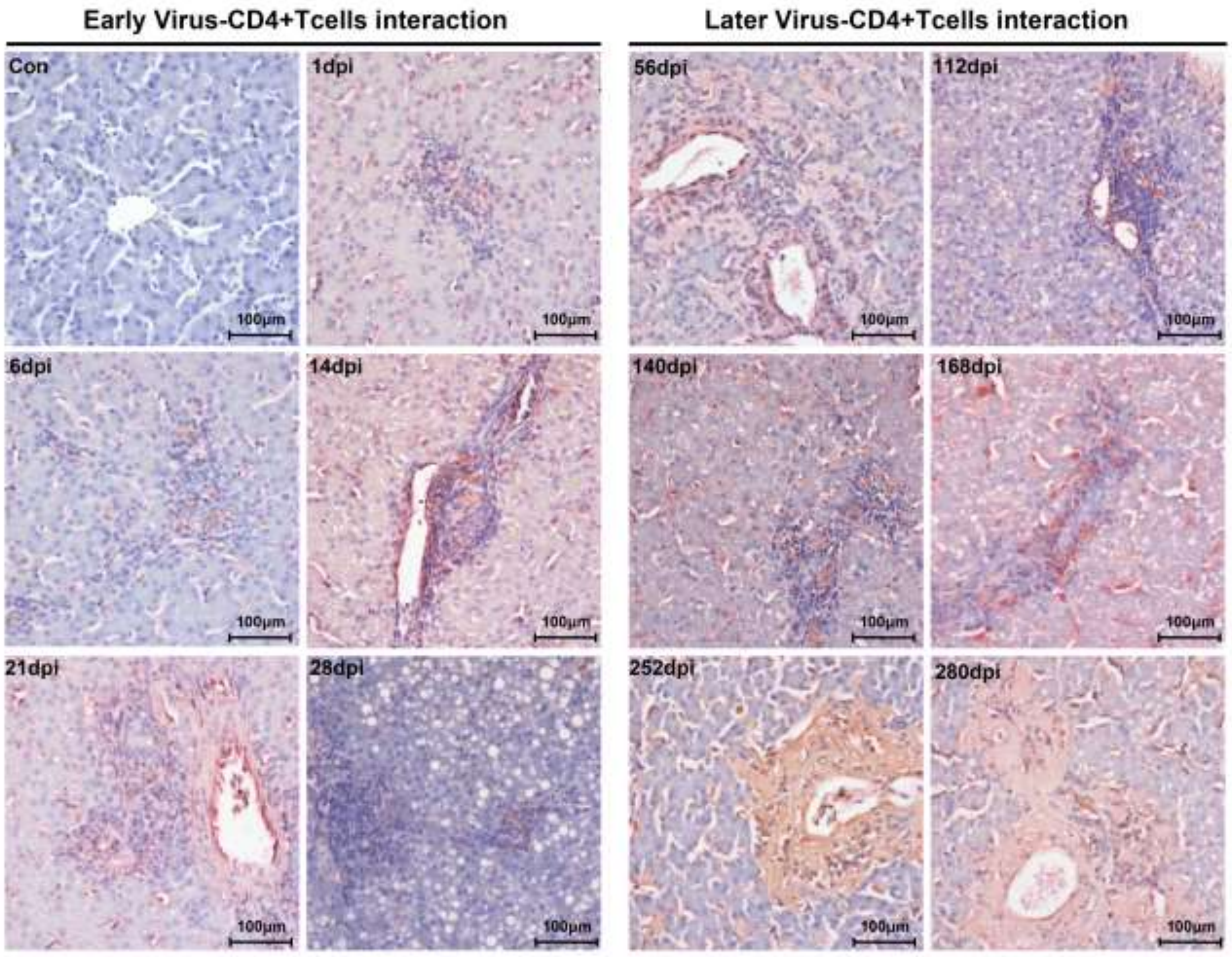

Supplementary Figure 2: Double staining of viral capsid and CD4+ positive T-cells in liver. Viral capsid and CD4+ T-cells were double stained by rabbit anti DHAV capsid antigen polyclonal antibody and mouse anti duck CD4 monoclonal antibody. Red colour and brown colour represent positive capsid antigens and CD4+ T-cells, respectively. (Con) represents liver without double primary antibody. The brightness and contrast are slightly modified to create a uniform background.

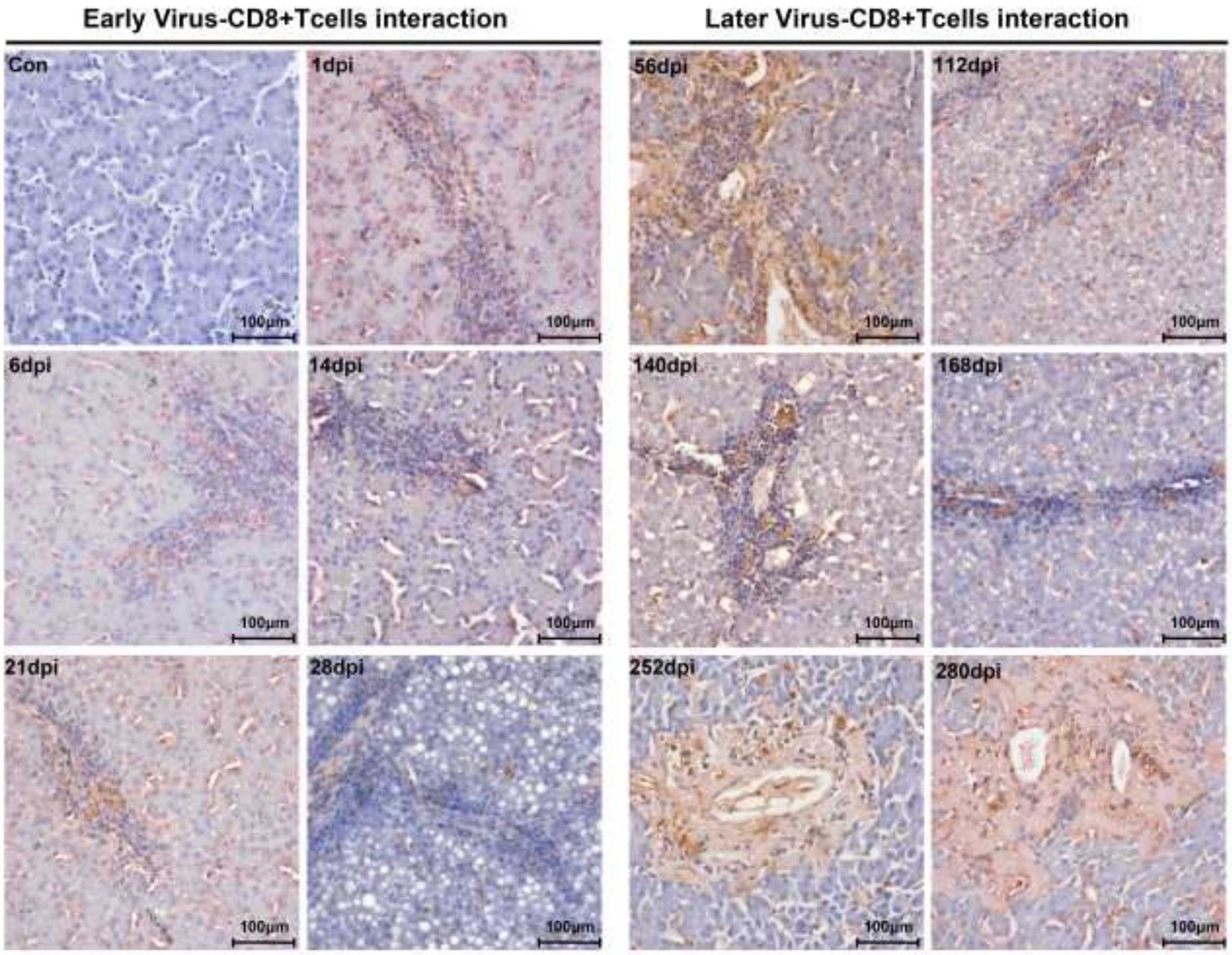

Supplementary Figure 3: Double staining of viral capsid and CD8+ positive T-cells in liver. Viral capsid and CD8+ T-cells were double stained with rabbit and DHAV capsid antigen polyclonal antibody and mouse anti duck CD8α monoclonal antibody. Red colour and brown colour represent positive capsid antigens and CD8α+ T-cells, respectively. (Con) represents liver without double primary antibody. The brightness and contrast are slightly modified to create a uniform background.

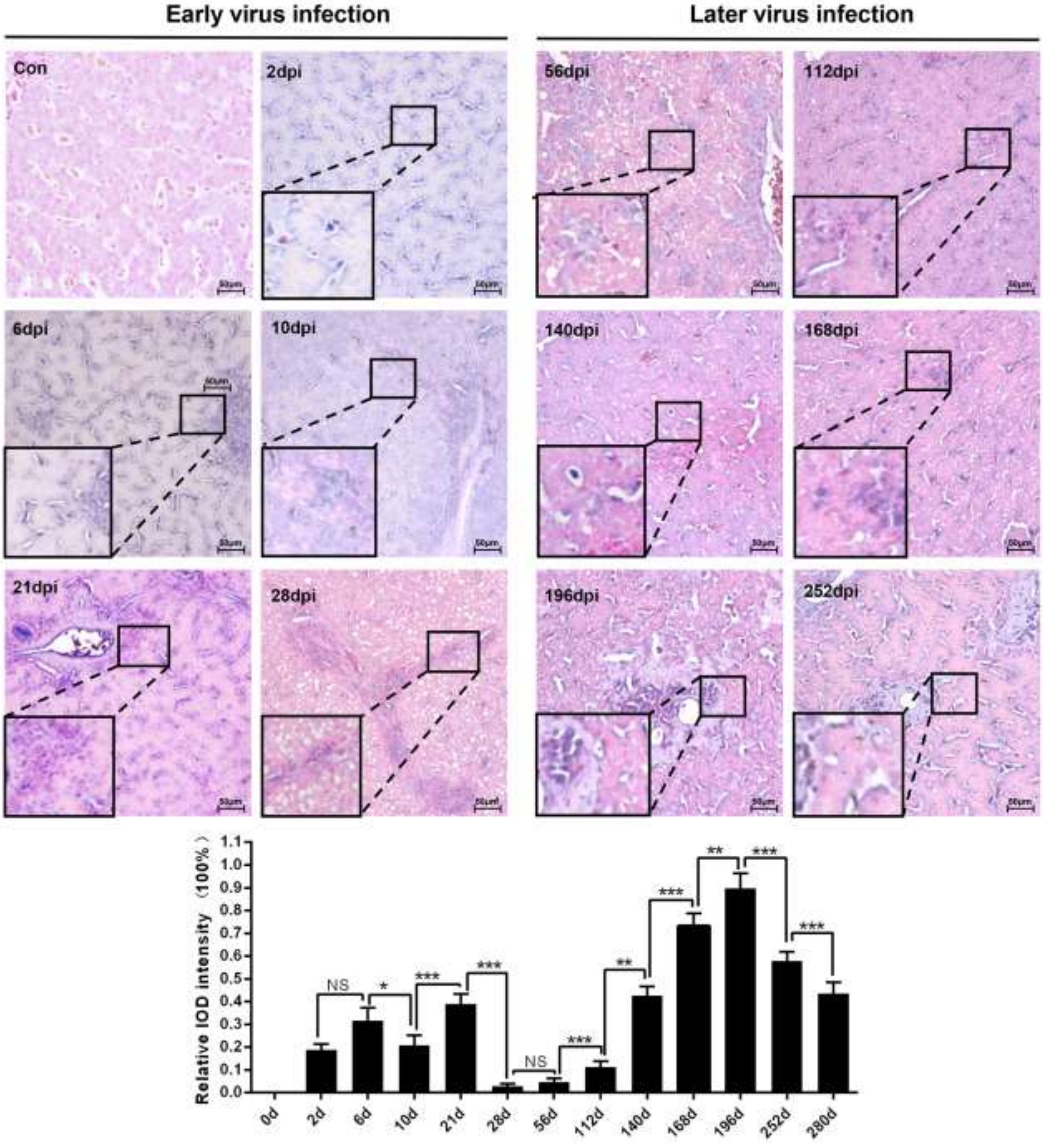

Supplementary Figure 4: Dynamic expression levels of viral capsid antigens in liver. Viral capsid is shown by rabbit and DHAV capsid antigen polyclonal antibody with blueviolet. 400× magnification of photograph matrices according to the time post-infection. Typical virus distribution was exemplified at the corner of each photograph. (Con) represents liver without primary antibody. The brightness and contrast are slightly modified to create a uniform background. The intensity of

positive staining was calculated by Image-Pro Plus software. The significant levels of their expression were analysed by Student T test. NS, No significance; *, P<0.05; **, P<0.01; ***, P<0.001.

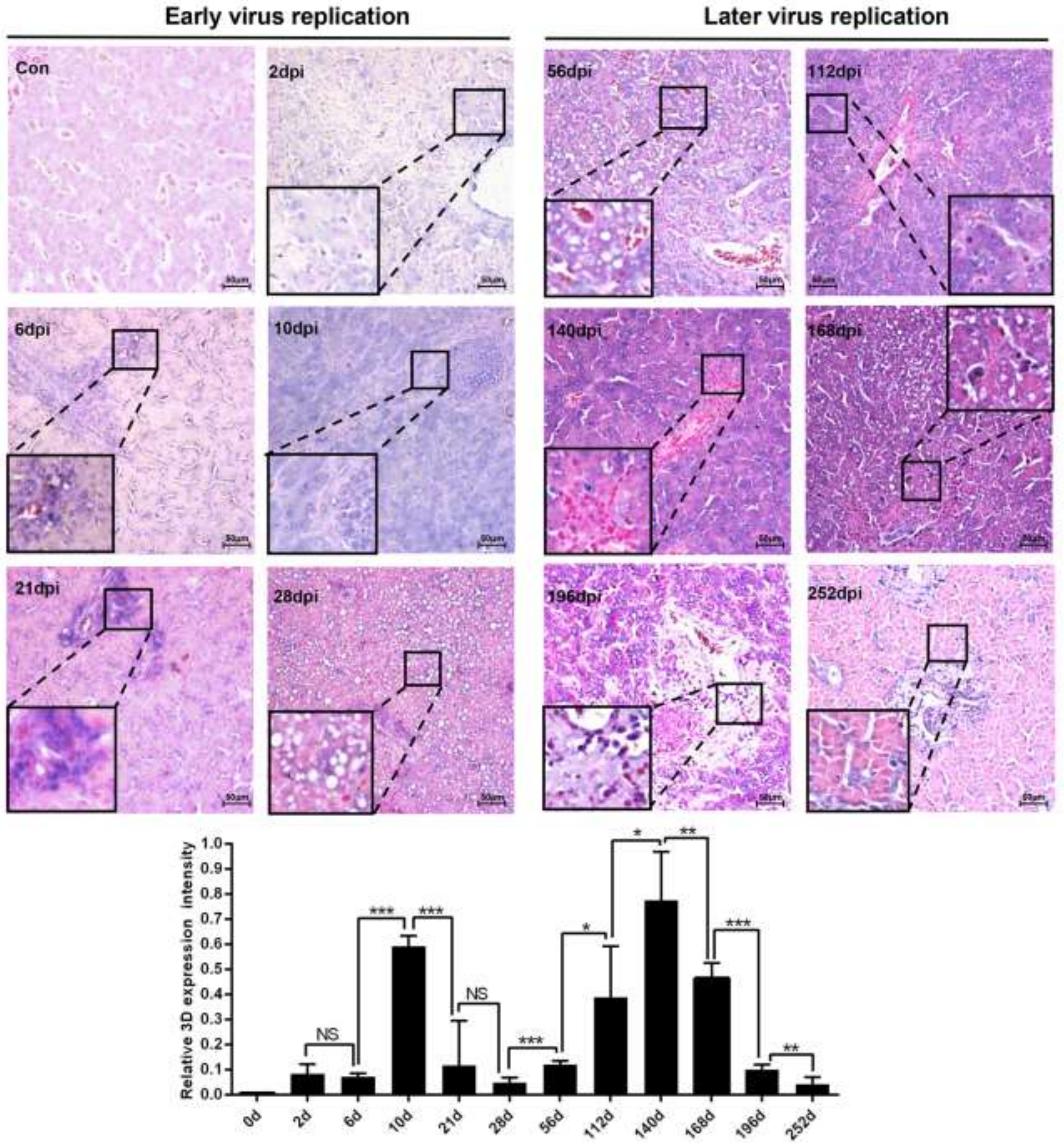

Supplementary Figure 5: Dynamic expression level of viral RNA polymerases in liver. Viral

replication was detected by rabbit anti DHAV RNA polymerase polyclonal antibody with blue-violet. (Con) represents liver without the primary antibody. The brightness and contrast are slightly modified to create a uniform background. The intensity of positive staining was calculated by Image-Pro Plus software. The significant levels of their expression were analysed by Student T test. NS, No significance; *, P<0.05; **, P<0.01; ***, P<0.001.

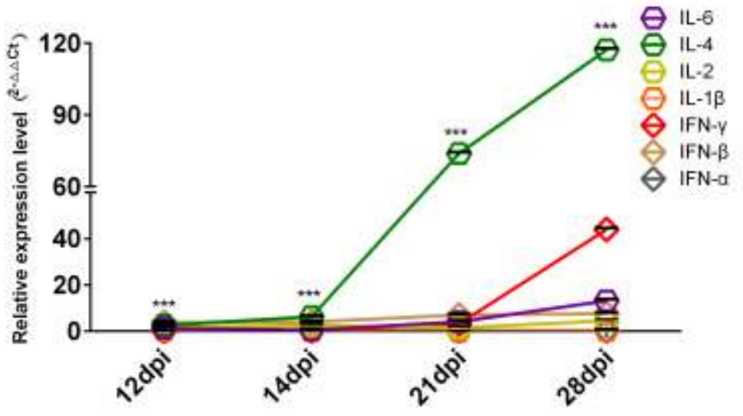

Supplementary Figure 6: Comparative analysis of interferons and interleukins from 12-dpi to 28dpi. The relative values for each cytokines were calculated by $2^{-\Delta\Delta Ct}$ method by comparing with the non-infected group. The significant difference at different time points between IFN-γ and IL-4 were calculated by student's T-test. *, P<0.05; **, P<0.01; ***, P<0.001.

Supplementary Table 1: Fold changes of immune related genes induced by DHAV-H strain ($2^{-\triangle\triangle Ct}$)

H	IFN-α	IFN-β	IFN-γ	IL-1β	IL-2	IL-4
0.5d	3.39±0.86	7.28±0.32	0.21±0.6	2.76±0.72	0.96±0.3	5.07±0.53
1d	0.62±0.77	6.48±0.29	9.89±0.39	0.92±0.5	0.67±0.38	3±0.51
2d	38.43±0.54	0.42±0.27	18.77±0.4	1.21±0.33	211.72±0.35	1792.5±0.55
6d	44.05±0.69	14.85±0.45	33.31±0.51	5.35±0.43	902.08±0.76	1413.7±0.67
8d	1.14±0.81	7.11±0.45	1.05±0.44	0.64±0.64	1.62±0.78	10.09±0.45
10d	0.21±0.85	0.01±0.34	0.23±0.64	0.57±0.67	0.6±0.39	0.66±0.45
12d	0.14±0.8	3.81±0.4	0.04±0.51	0.38±0.54	1.61±0.8	2.6±0.71
14d	0.17±0.6	4±0.4	0.07±0.42	0.25±0.5	2.57±0.48	6.28±0.5
21d	0.09±0.64	7.03±0.38	3.22±0.58	0.04±0.55	1.49±0.64	73.91±0.57
28d	0.25±0.55	7.65±0.42	44.09±0.61	0.27±0.65	4.53±0.73	117.24±0.71
H	IL-6	MHC-I	MHC-II	BAFF	CCL-19	CCL-21
0.5d	4.38±0.37	1.52±0.75	1.17±0.67	2.39±0.75	0.44±0.65	0.79±0.44
1d	1.05±0.72	1.08±0.65	0.56±0.85	0.6±0.5	0.5±0.65	0.23±0.67
2d	53.11±0.42	0.44±0.61	0.35±0.87	1.24±0.37	0.14±0.64	13.09±0.7
6d	50.39±0.27	2.15±0.48	0.5±0.71	0.75±0.6	0.37±0.83	16.99±0.69
8d	4.85±0.41	0.75±0.77	0.63±0.82	1.58±0.67	0.47±0.88	0.58±0.7
10d	0.09±0.79	0.36±0.6	0.45±0.71	0.42±0.72	0.58±0.86	0.67±0.76
12d	1.28±0.62	0.24±0.88	0.22±0.73	0.72±0.8	0.31±0.62	0.46±0.73
14d	0.54±0.57	0.24±0.44	0.09±0.51	0.5±0.44	0.23±0.67	0.48±0.69
21d	4.05±0.75	0.1±0.67	0.13±0.66	0.37±0.54	0.14±0.77	0.12±0.7
28d	13.21±0.74	0.24±0.87	0.2±0.62	0.44±0.52	0.13±0.72	0.14±0.76
H	TLR-7	TLR-3	β-defensin	RIG-1	MDA5	
0.5d	5.46±0.76	0.52±0.81	40.5±0.63	0.29±0.64	0.61±0.65	
1d	0.83±0.55	0.86±0.8	98.34±0.69	0.24±0.68	0.65±0.61	
2d	7.4±0.54	0.5±0.76	4.36±0.74	0.13±0.65	0.83±0.56	
6d	2.95±0.67	0.49±0.87	9.62±0.63	0.11±0.62	0.2±0.59	
8d	1±0.47	1.23±0.54	7.5±0.7	0.13±0.65	0.54±0.54	
10d	0.68±0.88	0.57±0.9	0.39±0.64	0.33±0.55	0.37±0.89	
12d	0.36±0.64	0.7±0.78	13.37±0.63	0.01±0.65	0.1±0.59	
14d	0.43±0.85	0.88±0.7	2.28±0.38	0.06±0.49	0.14±0.45	
21d	0.49±0.85	0.46±0.8	1.73±0.68	0.11±0.5	0.17±0.63	
28d	0.83±0.67	0.91±0.75	26.21±0.52	0.19±0.68	0.47±0.86	

Dynamic changes of virus load in liver

Supplementary Table 2: Dynamic changes of virus load in liver

dpi	Virus load（log10/g）	Precentage（%）
0	0	0
0.5	6.37±0.14	100.00%
1	7.18±0.32	100.00%
2	4.03±0.15	60.00%
4	6.03±0.56	100.00%
6	4.47±0.35	80.00%
8	5.50±0.30	80.00%
10	7.10±0.89	80.00%
12	6.84±0.30	100.00%
14	6.84±0.30	100.00%
21	3.93±0.56	100.00%
28	3.92±0.25	100.00%
56	5.91±0.17	100.00%
84	4.05±0.87	100.00%
112	4.02±0.23	50.00%
140	2.83±0.33	100.00%
168	3.60±0.45	60.00%
196	3.74±0.22	50.00%
224	2.67±0.00	25.00%
252	2.59±0.18	50.00%
280	0.00±0.00	0.00%

n=5

Members of picornavirus and fibrosis related genes in ducks and humans

Supplementary Table 3: Members of picornavirus and fibrosis related genes in ducks and humans

Picornaviridae	Accession No.	Fibrosis Related Genes	Accession No.	Comments
DHAV-H	JQ301467.1	Stromelysin-1	NP_002413.1	Homo Sapiens
DHAV-X	JQ316452.1	Stromelysin	AAB36942.1	Homo Sapiens
DHAV-ZJ	EF382778.1	MMP-3	AAA36321.1	Homo Sapiens
DHAV-R85952	NC_008250.2	Stromelysin-2	EOB07981.1	Anas platyrhynchos
Human parechovirus	JX575746.1	Stromelysin-1	XP_012961344.1	Anas platyrhynchos
Simian HAV	EU140838.1	Collagenase-3	XP_005010557.1	Anas platyrhynchos
HAV-HA120938	530669578	Interstitial collagenase	XP_005010575.1	Anas platyrhynchos
HA-HPA24A	M59810.1	Neutrophil collagenase	XP_011541138.1	Homo Sapiens
HAV	AF314208.1	Collagenase	AAB36941.1	Homo Sapiens
Human rhinovirus A	JN562722.1	Skin Collagenase	AAA35699.1	Homo Sapiens
Poliovirus	NC_002058.3	MMP-27	XP_005010555.1	Anas platyrhynchos
Triatoma virus	NC_003783.1	MMP-27	XP_011541252.1	Homo Sapiens
Porcine teschovirus	HQ020378.1	MMP-9	XP_012960610.1	Anas platyrhynchos
Equine rhinitis B virus	NC_003983.1	MMP-9	NP_004985.2	Homo Sapiens
Encephalomyelitis virus	KP892662.1	IV Collagenase	EOA96909.1	Anas platyrhynchos
Bovine Kobuvirus	NC_004421.1	IV Collagenase	NP_001121363.1	Homo Sapiens
FMDV	EF611987.1	Stromelysin-3	XP_005023630.2	Anas platyrhynchos
		MMP-28	XP_005010742.1	Anas platyrhynchos
		MMP-28	XP_011523533.1	Homo Sapiens
		MMP-18	Q99542.1	Homo Sapiens
		MMP-19	XP_011536661.1	Homo Sapiens
		MMP17	EOB02338.1	Anas platyrhynchos
		MMP-17	XP_011536659.1	Homo Sapiens
		MMP-25	XP_011520904.1	Homo Sapiens
		MMP-15	XP_005019998.1	Anas platyrhynchos
		MMP-16	XP_005016789.1	Anas platyrhynchos
		MMP-24	XP_005018109.1	Anas platyrhynchos
		MMP-24	XP_011526802.1	Homo Sapiens
		Hemopexin	EOA94121.1	Anas platyrhynchos
		Hemopexin	NP_000604.1	Homo Sapiens
		Vitronectin	XP_005025080.1	Anas platyrhynchos
		Glycoside hydrolase	AFR34918.1	Riemerella Anatipestifer

Primer sequences used in gene expression profiles

Supplementary Table 4: Primer sequences used in gene expression profiles

Gene	Forward primer	Reverse primer	AD	Reference
GAPDH	5’-ATGTTCGTGATGGGTGTGAA-3’	5’-CTGTCTTCGTGTGTGGCTGT-3’	AY436595	[2]
IFN-α	5’-TCCTCCAACACCTCTTCGAC-3’	5’-GGGCTGTAGGTGTGGTTCTG-3’	EF053034	[2]
IFN-β	5’-CCTCAACCAGATCCAGCATT-3’	5’- GGATGAGGCTGTGAGAGGAG-3’	AY831397	[2]
IFN-γ	5’-GCTGATGGCAATCCTGTTTT-3’	5’- GGATTTTCAAGCCAGTCAGC-3’	AJ012254	[2]
IL-1β	5’-TCGACATCAACCAGAAGTGC-3’	5’-GAGCTTGTAGCCCTTGATGC-3’	DQ393268	[2]
IL-2	5’-GCCAAGAGCTGACCAACTTC-3’	5’-ATCGCCCACACTAAGAGCAT-3’	AF294323	[2]
IL-4	5’-CCTCCACGGTTGTTTTCGAG-3’	5’-GTTGGAGGGTTCTGTGGAGG-3’	XM_005024359.1	[5]
IL-6	5’-TTCGACGAGGAGAAATGCTT-3’	5’-CCTTATCGTCGTTGCCAGAT-3’	AB191038	[2]
MHC-I	5’-GAAGGAAGAGACTTCATTGCCTTGG-3’	5’-CTCTCCTCTCCAGTACGTCCTTCC-3’	AB115246	[2]
MHC-II	5’-CCACCTTTACCAGCTTCGAG-3’	5’-CCGTTCTTCATCCAGGTGAT-3’	AY905539	[2]
BAFF	5’-TGTGCACGTCATCCAACAGA-3’	5’-GCCACAGGAATGTGACAGGA-3’	DQ445092	[5]
CCL21	5’-GGAGAAGCAGAAGAACCCCC-3’	5’-GGGAAAGCATCCGTCCTCTC-3’	DR764376	[5]
CCL19	5’-CCAGGAAGGTCCCAAATAAA-3’	5’-GTAGTAGGAGGTGGAAGCAAGTC-3’	DR766004	[5]
β-defensin	5’-CCAGGTTTCTCCAGGATTGT-3’	5’-AACCCAAAGCAACTTCCAAC-3’	AY641439	[5]
TLR7	5’-CCTTTCCCAGAGAGCATTCA-3’	5’-TCAAGAAATATCAAGATAATCACATCA -3’	AY940195	[2]
TLR3	5’-AACACTCCGCCTAAGTATCAT-3’	5’-CTATCCTCCACCCTTCAAAA-3’	JN573268	[5]
RIG-1	5’-GCGTACCGCTATAACCCACA-3’	5’-CCTTGCTGGTTTTGAACGC-3’	AB772012.1	[5]
MDA5	5’-GCTGAAGAAGGCCTGGACAT-3’	5’-TCCTCTGGACACGCTGAATG-3’	KJ451070.1	[5]

References

1. Yang M, Cheng A, Wang M, Xing H. Development and application of a one-step real-time Taqman RT-PCR assay for detection of Duck hepatitis virus type1. J Virol Methods. 2008; 153: 55-60. doi: 10.1016/j.jviromet.2008.06.012.
2. Adams SC, Xing Z, Li J, Cardona CJ. Immune-related gene expression in response to H11N9 low pathogenic avian influenza virus infection in chicken and Pekin duck peripheral blood mononuclear cells. Mol Immunol. 2009; 46: 1744-9. doi: 10.1016/j.molimm.2009.01.025.
3. Tamura K, Stecher G, Peterson D, Filipski A, Kumar S. MEGA6: Molecular Evolutionary Genetics Analysis version 6.0. Mol Biol Evol. 2013; 30: 2725-9. doi: 10.1093/molbev/mst197.
4. Livak KJ, Schmittgen TD. Analysis of Relative Gene Expression Data Using Real-Time Quantitative PCR and the $2^{-\Delta\Delta Ct}$Method. methods. 2001; 25: 402-8. doi: 10.1006/meth.2001.1262.
5. Ou X, Mao S, Jiang Y, Zhang S, Ke C, Ma G, Cheng A, Wang M, Zhu D, Chen S, Jia R, Liu M, Sun K, et al. Viral-host interaction in kidney reveals strategies to escape host immunity and persistently shed virus to the urine. Oncotarget. 2016; 8: 7336-49. doi: 10.18632/oncotarget.14227.
